# Inhibitory Effects of *Saururus chinensis* Extract on Receptor for Advanced Glycation End-Products-Dependent Inflammation and Diabetes-Induced Dysregulation of Vasodilation

**DOI:** 10.3390/ijms23105757

**Published:** 2022-05-20

**Authors:** Kenjiro Hayashi, Koichi Sato, Seishi Ochi, Shuhei Kawano, Seiichi Munesue, Ai Harashima, Yu Oshima, Kumi Kimura, Takashi Kyoi, Yasuhiko Yamamoto

**Affiliations:** 1 Food Development Labs, Functional Food Division, Nippon Shinyaku Co., Ltd., Kyoto 601-8550, Japan; ke.hayashi@po.nippon-shinyaku.co.jp (K.H.); ko.sato@po.nippon-shinyaku.co.jp (K.S.); s.ochi@po.nippon-shinyaku.co.jp (S.O.); sh.kawano@po.nippon-shinyaku.co.jp (S.K.); t.kyoi@po.nippon-shinyaku.co.jp (T.K.); 2 Department of Biochemistry and Molecular Vascular Biology, Kanazawa University Graduate School of Medical Sciences, Kanazawa 920-8640, Japan; smunesue@med.kanazawa-u.ac.jp (S.M.); aharashima@staff.kanazawa-u.ac.jp (A.H.); cantabile.6102@gmail.com (Y.O.); kukimura@staff.kanazawa-u.ac.jp (K.K.)

**Keywords:** receptor for advanced glycation end-products (RAGE), *Saururus chinensis* (Lour.) Baill, vascular relaxation

## Abstract

Advanced glycation end-products (AGEs) and the receptor for AGEs (RAGE) are implicated in inflammatory reactions and vascular complications in diabetes. Signaling pathways downstream of RAGE are involved in NF-κB activation. In this study, we examined whether ethanol extracts of *Saururus chinensis* (Lour.) Baill. (SE) could affect RAGE signaling and vascular relaxation in streptozotocin (STZ)-induced diabetic rats. Treatment with SE inhibited AGEs-modified bovine serum albumin (AGEs-BSA)-elicited activation of NF-κB and could compete with AGEs-BSA binding to RAGE in a dose-dependent manner. Tumor necrosis factor-α (TNF-α) secretion induced by lipopolysaccharide (LPS)—a RAGE ligand—was also reduced by SE treatment in wild-type *Ager^+/+^* mice as well as in cultured peritoneal macrophages from *Ager*^+/+^ mice but not in *Ager*^−/−^ mice. SE administration significantly ameliorated diabetes-related dysregulation of acetylcholine-mediated vascular relaxation in STZ-induced diabetic rats. These results suggest that SE would inhibit RAGE signaling and would be useful for the improvement of vascular endothelial dysfunction in diabetes.

## 1. Introduction

Advanced glycation end-products (AGEs) are non-enzymatically generated from amino residues of proteins and reducing sugars through the Maillard reaction [1]. AGEs are known to cause diabetic vascular complications and atherosclerosis [2,3,4]. The receptor for AGEs (RAGE) can bind AGEs [5] and is now recognized as a pattern-recognition receptor expressed on the plasma membrane; it also binds other ligands such as S100 protein, high mobility group box (HMGB)-1, and lipopolysaccharides (LPS) [6,7,8]. These ligands activate NF-κB signaling through RAGE and lead to inflammatory reactions, foam cell formation, and atherosclerosis [9,10,11]. We recently reported that oxytocin is a ligand associated with RAGE, but it cannot cause any activation or inhibition of the post-receptor signaling pathways or compete with other ligands that bind to RAGE [12,13]. Thus, RAGE is recognized as an oxytocin passive transporter on the blood-brain barrier [13,14]. In contrast, ligand-RAGE-NF-κB signaling could increase the expression of vascular cell adhesion molecule-1 (VCAM-1), intracellular adhesion molecule-1 (ICAM-1), endothelin 1, and E-selectin in vascular endothelial cells and tumor necrosis factor-α (TNF-α) in macrophages, which are involved in atherosclerosis progression [15,16,17]. In addition, RAGE signaling could reduce phosphorylation of serine residues of endothelial NO synthase (eNOS), resulting in the deactivation of this enzyme and impairment of endothelial function [18]. Furthermore, diabetic double knockout mice lacking RAGE and apolipoprotein E (ApoE) (*Ager*^−/−^/*ApoE*^−/−^ mice) have lower atherosclerotic plaque areas than diabetic *Ager*^+/+^/*ApoE*^−/−^ mice [19]. Thus, RAGE plays a central role in the development of vascular derangements associated with diabetes. Considering this fact, strategies targeting RAGE would be useful for the prevention and treatment of vascular dysfunction and atherosclerosis.

To develop RAGE inhibitors, we screened approximately 600 botanical extracts using stably transfected rat C6 glioma cells that expressed human full-length RAGE and the NF-κB enhancer-luciferase system [11,20] and recently succeeded in identifying the candidates (unpublished data). Among them, *Saururus chinensis* leaf extract, the most potent candidate, could inhibit RAGE-dependent NF-κB activation. *Saururus chinensis* belongs to the family Saururaceae and is found in East Asia, including South Korea, China, and Japan. *Saururus chinensis* leaves have been used for patients with edema, pneumonia, and inflammation diseases as a traditional medicine [21], which has an anti-inflammatory effect via downregulation of NF-κB signaling [22]. *Saururus chinensis* leaves contain essential oil components such as methylnonylketone and various lignans such as sauchinone, saucerneol, and manassantin [23,24]; these components have been shown to have anti-inflammatory effects [24,25,26].

The present study attempted to prove the beneficial effects of ethanol extracts of *Saururus chinensis* (Lour.) Baill. (SE), which was identified through our botanical natural material pre-screening on inhibiting RAGE signaling and inflammation and improving the impairment of acetylcholine-induced vasodilation of vascular endothelial function in diabetes.

## 2. Results

### 2.1. Inhibitory Effects of Saururus chinensis Extracts on RAGE Signaling

**Figure 1:**
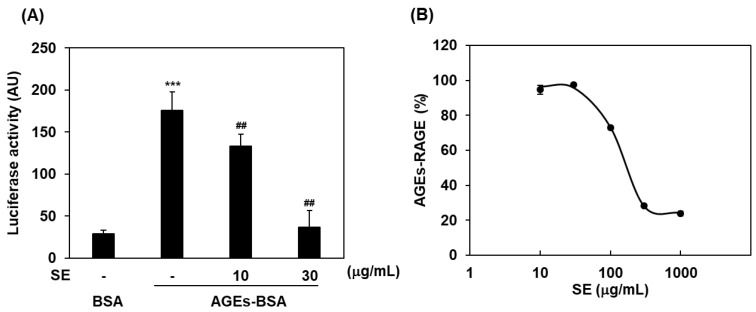
Inhibitory effects of SE on RAGE intracellular signaling and AGEs–RAGE interactions. (**A**) RAGE-dependent NF-κB activity assay. Glyceraldehyde-derived AGEs-BSA (AGEs-BSA) (100 μg/mL), non-glycated BSA control (BSA), and SE (10 or 30 μg/mL) were used. Data are expressed as mean ± SD (n = 6). ***, *p* < 0.001 vs. BSA; ^##^, *p* < 0.01 vs. AGEs-BSA only (-). (**B**) AGEs–RAGE-binding plate-competition assay by SE. SE (10, 30, 100, 300, or 1000 μg/mL) was incubated with esRAGE (2.0 μg/mL). Data are expressed as mean ± SD (n = 3). BSA, bovine serum albumin; SE, ethanol extracts of *Saururus chinensis* (Lour.) Baill.; AGEs-BSA, AGEs-modified bovine serum albumin; AU, arbitrary unit.

To assess RAGE-dependent NF-κB activation or its inhibition, we used C6 glioma cells expressing human full-length RAGE and a firefly luciferase reporter gene under the control of the NF-κB promoter [20]. When cells were exposed to glyceraldehyde-derived AGEs-BSA, NF-κB activity was significantly increased in comparison with that in the non-glycated BSA control (Figure 1A). In the presence of SE, the elevation of AGEs-BSA-induced NF-κB activation was dose-dependently and significantly suppressed (10 and 30 μg/mL; *p* < 0.01; Figure 1A). We next examined whether AGEs-BSA binding to RAGE could be affected by SE using a plate-binding assay. Purified recombinant human endogenous secretory RAGE (esRAGE), which possesses a ligand-binding extracellular domain of RAGE, was used. SE was found to compete in a dose-dependent manner for the interaction between AGEs-BSA and esRAGE (Figure 1B).

### 2.2. Inhibitory Effects of Saururus chinensis Extracts on LPS-Induced Cytokine Release

**Figure 2:**
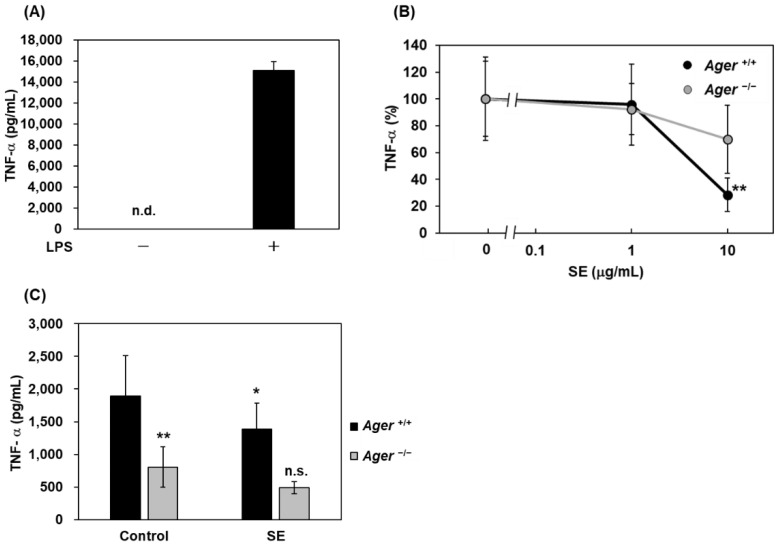
Inhibitory effects of SE on LPS-induced RAGE-dependent TNF-α secretion. (**A**) Mouse macrophages from *Ager*^+/+^ mice were incubated with or without LPS (50 ng/mL). TNF-α concentrations in cultured media were measured using ELISA. Data are expressed as mean ± SD (n = 4). n.d.; not detected. (**B**) Mouse peritoneal macrophages from *Ager*^+/+^ or *Ager*^−/−^ mice were pre-incubated with SE (0, 1 or 10 μg/mL) for 1 h before stimulation with LPS (50 ng/mL) for 24 h. TNF-α concentrations in cultured media were measured using ELISA. Data are expressed as mean ± SD (n = 3–4). **, *p* < 0.01 vs. LPS only (0). (**C**) TNF-α levels in the plasma of LPS-induced septic mice. *Ager*^+/+^ or *Ager*^−/−^ mice were orally administered with SE (100 mg/kg) before intraperitoneal injection with LPS (1 mg/kg). Plasma TNF-α concentrations were measured using ELISA. Data are expressed as mean ± SD (n = 6–17). *, *p* < 0.05 vs. control without SE treatment; **, *p* < 0.01 vs. *Ager*^+/+^ control; n.s. indicates no significance vs. *Ager*^−/−^ control. SE, ethanol extracts of Saururus chinensis (Lour.) Baill.; LPS, lipopolysaccharide; TNF-α, tumor necrosis factor-α.

We previously reported that LPS could directly bind RAGE and then induce NF-κB activation, leading to TNF-α release in macrophages isolated from wild-type *Ager^+/+^* mice [8]. Accordingly, we next addressed the question of whether SE could inhibit LPS-induced TNF-α secretion in mouse peritoneal macrophages. We observed LPS-stimulated TNF-α secretion in peritoneal macrophages from wild-type *Ager*^+/+^ mice (Figure 2A), and the elevation of TNF-α levels was significantly suppressed by the addition of SE at 10 μg/mL in *Ager^+/+^* mouse peritoneal macrophages (*p* < 0.01), but not in *Ager*^−/−^ (Figure 2B). SE-mediated suppression of TNF-α secretion from *Ager*^−/−^ macrophages was lesser than that from *Ager^+/+^* macrophages, supporting the dependency of RAGE signaling. Furthermore, we evaluated the effects of SE using an LPS-loaded septic model. Oral administration of SE (100 mg/kg) significantly suppressed LPS-induced TNF-α elevation in the plasma of *Ager^+/+^* mice (*p* < 0.05; Figure 2C). However, in *Ager*^−/−^ mice, plasma TNF-α levels after intraperitoneal LPS loading were significantly lower than those in *Ager^+/+^* mice, and SE did not affect the plasma TNF-α levels in LPS-injected *Ager*^−/−^ mice (Figure 2C).

### 2.3. Effects of Saururus chinensis Extracts on Impairment of Acetylcholine-Induced Vasodilatation in Diabetes

**Figure 3:**
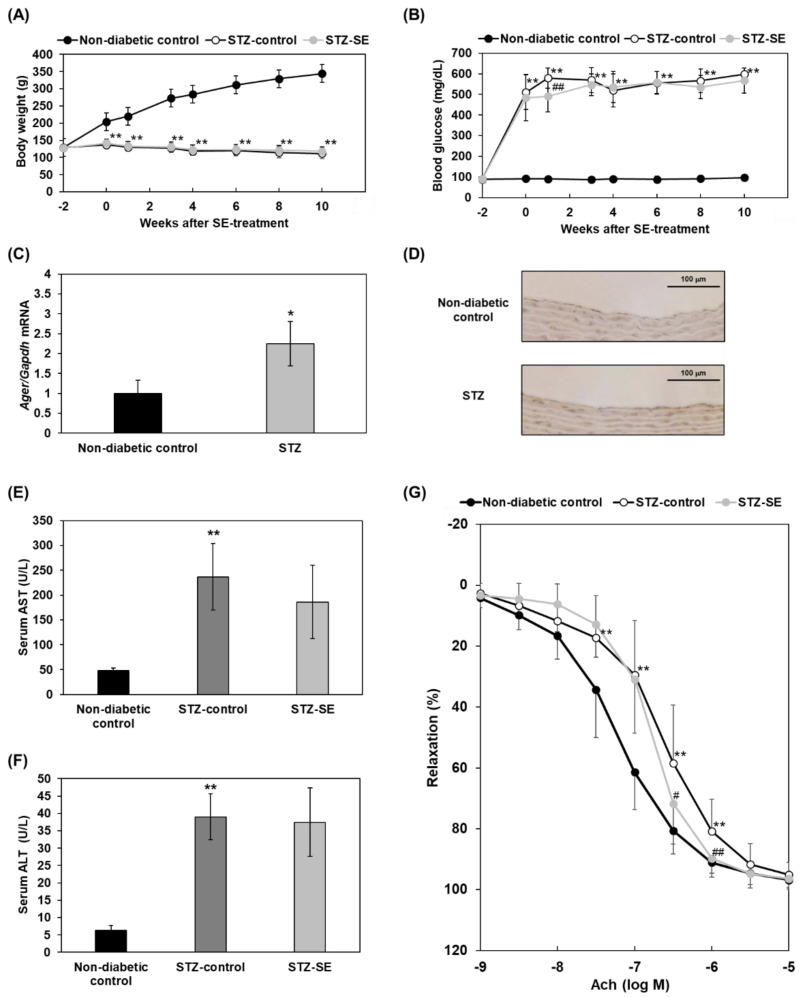
Beneficial effects of SE on vascular relaxation in STZ-induced diabetic rats. Rats were intraperitoneally injected with STZ (50 mg/kg), and the STZ-SE group was treated with 0.1% SE-mixed chow for 10 weeks. (**A**) Body weights. Data are expressed as mean ± SD (n = 6–9). **, *p* < 0.01 vs. Non-diabetic control. (**B**) Blood glucose levels. Data are expressed as mean ± SD (n = 6–9). **, *p* < 0.01 vs. Non-diabetic control. ^##^, *p* < 0.01 vs. STZ-control. (**C**) *Ager* mRNA expression in aortas at 10 weeks after STZ administration by qPCR. Data are expressed as mean ± SD (n = 3). *, *p* < 0.05 vs. Non-diabetic control. (**D**) RAGE protein expression in endothelia of the thoracic aortas were observed by immunohistochemistry. (**E**) Serum AST levels. Data are expressed mean ± SD (n = 6–9). **, *p* < 0.01, vs. Non-diabetic control. (**F**) Serum ALT levels. Data are expressed mean ± SD (n = 6–9). **, *p* < 0.01, vs. Non-diabetic control. (**G**) Vascular relaxation. Cumulative concentrations of acetylcholine (10^−9^ to 10^−5^ M) were added to the chamber, and the tension was recorded. Data are expressed as mean ± SD (n = 6–9). **, *p* < 0.01 vs. Non-diabetic control. ^#^, *p* < 0.05, vs. STZ-control; ^##^, *p* < 0.01 vs. STZ-control. STZ-SE, SE-treated and STZ-induced rats; Ach, acetylcholine; Gapdh, Glyceraldehyde-3-phosphate dehydrogenase; AST, aspartate aminotransferase; ALT, alanine aminotransferase.

To explore the functional effects of SE on vascular physiological responses, we used thoracic aortic rings to assess acetylcholine-induced vascular relaxation. A previous report showed that acetylcholine-induced endothelium-mediated aortic vasodilatation is impaired in diabetic animals, possibly due to endothelial dysfunction and vascular inflammation. To induce vascular damage in diabetes, we used male streptozotocin (STZ)-induced diabetic rats as well as non-diabetic controls. After the induction of diabetes, the body weights of STZ rats were significantly lower than those of non-diabetic controls during the observation period (Figure 3A), and SE treatment did not change body weight in STZ-induced diabetic rats (Figure 3A). Blood glucose levels were significantly elevated after STZ injection in rats (*p* < 0.01; Figure 3B). However, 10-week SE treatment in STZ-induced diabetic rats demonstrated almost no effects on lowering blood glucose levels; the rats showed significantly lower levels of blood glucose only 1 week after starting treatment (*p* < 0.01; Figure 3B). The expressions of *Ager* mRNA (RAGE gene) in aortas and of RAGE protein in endothelia of the thoracic aortas were increased (Figure 3C,D). SE administration did not change aspartate serum transferase (AST) and alanine amino transferase (ALT) in diabetic groups, suggesting no obvious multiorgan damages (Figure 3E,F); this is compatible with a previous report [27]. At 10 weeks of SE treatment in diabetic rats, thoracic aortic rings were prepared and subjected to relaxation responses to cumulative concentrations of acetylcholine (10^−9^ to 10^−5^ M) to compare their responses with those in non-treated STZ rats or non-diabetic controls. In aortas from all animal groups, acetylcholine (10^−9^ to 10^−5^ M) caused concentration-dependent relaxation in aortas with 100% relaxation at 10^−5^ M (Figure 3G). The acetylcholine-induced relaxation of aortic rings from STZ-induced diabetic rats was significantly smaller at concentrations of 10^−7.5^ to 10^−6^ M than that observed in non-diabetic controls (Figure 3G). A marked rightward shift of the concentration-response curve to acetylcholine (EC50) was observed in the STZ-induced diabetic group compared to the non-diabetic control group (Figure 3G). Treatment with SE in diabetic rats showed a significant improvement in the deteriorated acetylcholine-induced relaxation response at 10^−6.5^ and 10^−6^ M (Figure 3G).

## 3. Discussion

In the present study, we found that SE, which could be a potential botanical extract candidate identified through our initial screening, dose-dependently inhibited AGEs-BSA-induced NF-κB activation in C6 glioma cells (Figure 1A) and competed with AGEs-BSA binding for the extracellular domain of RAGE (Figure 1B). These results suggest that SE acts as an antagonist by inhibiting AGEs-BSA binding to RAGE and blocking the subsequent RAGE signaling of NF-κB activation. We previously reported that LPS could be directly associated with RAGE and induce inflammatory reactions such as TNF-α production in a RAGE-dependent manner [8]. In this study, we found that SE supplementation suppressed LPS-induced TNF-α secretion in *Ager^+/+^* mouse peritoneal macrophages but not in *Ager*^−/−^ (Figure 2B) and significantly blocked plasma TNF-α elevation in an LPS-injected septic model of *Ager^+/+^* mice (Figure 2C). Plasma TNF-α elevation by LPS injection was found to be lower than that in wild-type *Ager^+/+^* mice, and TNF-α levels were not reduced after SE treatment in *Ager*^−/−^ mice (Figure 2C). We speculate that SE could not attenuate LPS-RAGE-independent TNF-α elevation in plasma, which might be driven by toll-like receptor 4 (TLR4) signaling [8] (Figure 2C).

We previously reported that low molecular weight heparin (LMWH) exhibits a competitive inhibitory action on AGEs-BSA binding to RAGE and an antagonistic effect on AGE–RAGE signaling [20]. In addition, LMWH significantly inhibited NF-κB activation induced by the RAGE ligand HMGB1 [28]. In addition, we recently identified papaverine, an opium alkaloid antispasmodic drug used for visceral spasm and vasospasm, as a RAGE inhibitor using the conversion to small molecules through an optimized-peptide strategy drug design system [29]. Papaverine was found to inhibit RAGE-dependent NF-κB activation driven by HMGB1 [29]. These collective data with the findings of this study allow us to interpret that the mode of action of SE is common to all inhibitors against RAGE; this mode involves the antagonistic inhibition by binding SE to the V domain of extracellular RAGE and blocking the binding of other ligands to RAGE, leading to silencing of subsequent RAGE signaling.

Next, we evaluated the beneficial and functional effects of SE on vascular relaxation in STZ-induced diabetic rats. Treatment with SE ameliorated the impairment of responsiveness to acetylcholine in vascular relaxation in diabetes (Figure 3G). Previous reports demonstrated that endothelium-dependent vascular relaxation induced by acetylcholine in the aorta was attenuated in STZ-induced diabetic rats [30,31]. Several underlying causes of diabetic endothelial dysfunction in the model of acetylcholine-induced vascular relaxation are known: (1) production of reactive oxygen species (ROS) by hyperglycemia per se, which reduces nitric oxide (NO) bioavailability; (2) higher levels of plasma TNF-α which induce vascular inflammation; (3) TNF-α, which directly induces endothelial dysfunction via NF-κB activation, impairment of eNOS expression, and ROS formation; and (4) the AGEs–RAGE axis [30,31,32,33,34,35,36]. For the AGEs–RAGE axis, the treatment of a chemical antagonist, FPS-ZM1, against RAGE or soluble RAGE, a decoy form against signaling-yielding membrane-bound full-length RAGE, could restore endothelium-mediated vascular relaxation of aortas in response to acetylcholine in aged rats [35,36]. Additionally, it is reported that increased staining for both AGEs and RAGE was detected in the aorta of STZ-induced diabetic rats, and diabetes-induced aortic stiffening and cardiac hypertrophy were prevented by an inhibitor of AGEs [37]. These results are compatible with our findings in this study. Thus, RAGE inhibition could be beneficial in the impairment of vascular endothelial function in diabetes. Considering the equivalent dose calculation based on body surface area, a SE dose of 100 mg/kg in mice (approximately 20 g) is equivalent to a dose of 8.1 mg/kg for humans (approximately 60 kg and 480 mg/day/person). In addition, a 0.1% SE-mixed diet for a rat (approximately 120 g) is equivalent to a dose of 166 mg/kg/day since the food intake is around 20 g/day in a rat. Considering the calculation, SE 166 mg/kg/day is equivalent to 27 mg/kg/day for humans (approximately 60 kg and 1600 mg/day/person), which may be within the permissive range for humans to intake.

In conclusion, SE could be a potent RAGE inhibitor for the prevention and treatment of diabetes-associated endothelial dysfunction seen in the early phase of developing atherosclerosis and other RAGE-associated diseases such as inflammation. Further studies are required to identify the active pure component in SE and to perform the experiments to check the safety of SE and its efficacy and effectiveness in comparison with existing drugs such as non-steroidal anti-inflammatory drugs (NSAIDs).

## 4. Materials and Methods

### 4.1. Sample Preparation

*Saururus chinensis* (Lour.) Baill. is native to China. *Saururus chinensis* stems and leaves were harvested from July to September and sun-dried in Hubei Province of China in 2019. The dried materials were imported and maintained at room temperature. The dried materials were ground using a blender (Osaka Chemical, Osaka, Japan). The powdered sample (4 kg) was added to ethanol (40 L) and then extracted at 80 °C for 60 min. The mixture was filtered, and the filtrates were concentrated under reduced pressure. The dried residues were obtained as SE.

### 4.2. Animals

*Ager*^−/−^ mice (C57BL/6 background) were generated as previously described [20], and *Ager^+/+^* mice (C57BL/6) were purchased from Charles River Laboratories Japan, Inc. Wistar rats were purchased from Japan SLC Inc (Hamamatsu, Japan). Mice and rats at 10 and 5 weeks of age, respectively, were used for the experiments.

### 4.3. LPS-Induced Septic Mouse Model

*Ager^+/+^* and *Ager*^−/−^ mice were orally administered SE (100 mg/kg) or 10% dimethyl sulfoxide (DMSO) as a negative control before intraperitoneal injection with lipopolysaccharide (LPS; 055:B5 from *E. coli*) (1 mg/kg) (Sigma-Aldrich, Tokyo, Japan). Blood was collected from the tail vein 1 h after LPS injection.

### 4.4. STZ-Induced Diabetic Rat Model

Male Wistar rats were intraperitoneally injected with STZ (50 mg/kg) dissolved in 0.5 M citrated buffer (pH 4.5). Two weeks after injection, blood glucose levels in the tail vein were measured using LAB Gluco (Research & Innovation Japan Inc., Chiba, Japan). Diabetic rats (blood glucose > 300 mg/dL) were randomly divided into two groups (control and SE-treated). The SE-treated group was fed 0.1% SE-mixed chow for 10 weeks. Body weight and blood glucose levels were measured every 1–2 weeks. Ten weeks after the induction of diabetes, thoracic aortas were isolated from rats under isoflurane anesthesia.

### 4.5. NF-κB Luciferase Assay

Rat C6 glioma cells expressing human full-length RAGE and a firefly luciferase reporter gene were used for this assay, as previously described [20]. Briefly, C6 cells were incubated for 4 h in Dulbecco’s modified Eagle’s medium supplemented with 0.1% FBS and then stimulated with glyceraldehyde-derived AGEs-BSA (100 μg/mL) with or without SE (10 and 30 μg/mL) for 4 h [38]. Luciferase activity was assayed using the Luciferase Assay System (Promega Corporation, Madison, WI, USA), and luminescence was measured using a GloMax^®^ Navigator Microplate Luminometer (Promega Corporation, Madison, WI, USA).

### 4.6. Plate-Binding Assay

The SE competition assay was performed using an AGEs-BSA-coated plate as previously described [20]. Briefly, human esRAGE (2.0 μg/mL) was incubated with SE (10, 30, 100, 300, and 1000 μg/mL) at room temperature (RT) for 1 h on AGEs-BSA-coated plates. After incubation, the plate was washed three times with 0.01% Tween-20, 0.15 M NaCl, and 20 mM Tris-HCl (pH 7.5), horseradish peroxidase (HRP)-labeled anti-RAGE antibody (B-Bridge International, Inc., Santa Clara, CA, USA) was added, and the plate was incubated at RT for 1 h. The plates were washed, the substrate of HRP was added, and the absorbance at 450 nm was measured using a microplate reader (Hitachi High-Tech Science Corporation, Tokyo, Japan).

### 4.7. Real-Time Reverse Transcription PCR

Total RNA was extracted from thoracic aortas using TriPure Isolation Reagent (Roche, Mannheim, Germany). cDNA was synthesized using a High-Capacity cDNA Reverse Transcription Kit (Thermo Fisher Scientific, Waltham, MA, USA). Real-time PCR was performed using TB Green^®^ Premix Ex Taq™ II (Tli RNaseH Plus) (Takara Bio Inc., Shiga, Japan). The rat *Ager* primer sequence was 5′- CCTGAGACGGGACTCTTCACGCTTCGG-3′ (forward) and 5′-CTCCTCGTCCTCCTGGCTTTCTGGGGC-3′ (reverse), and the rat *Gapdh* primer sequence was 5′-TGCCACTCAGAAGACTGTGG-3′ (forward) and 5′-TTCAGCTCTGGGATGACCTT-3′ (reverse).

### 4.8. Immunohistochemistry

Rat thoracic aorta samples were stored in 10% neutral buffered formalin, paraffin-embedded, and sectioned at 3 μm. The samples were antigen-activated by microwave heat treatment, followed by the inactivation of endogenous peroxidase with hydrogen peroxide. Then, blocking was performed using protein block serum-free (Dako Denmark A/S, Kopenhagen, Denmark), and the samples were reacted with a RAGE Polyclonal Antibody (Bioss Inc., Boston, MA, USA) (dilution: 1:500) and Histofine Simple Stain Rat MAX-PO (R) (NICHIREI BIOSCIENCE INC., Tokyo, Japan). Substrate for DAB was then added.

### 4.9. Isolation of Peritoneal Macrophages

Peritoneal macrophages were collected as described previously [39]. Briefly, *Ager^+/+^* or *Ager*^−/−^ mice were intraperitoneally injected with 1 mL 4% Brewer thioglycollate medium, and peritoneal macrophages were collected. The collected cells were washed with Roswell Park Memorial Institute (RPMI) 1640 medium supplemented with 10% FBS and seeded into 24 well-plates. After 18–20 h incubation, the supernatant was changed to RPMI1640 serum-free media and incubated for 4 h. Cells were pre-incubated with SE (1.0 or 10 μg/mL) for 1 h before stimulation with LPS (055:B5 from *E. coli*; Sigma-Aldrich, Tokyo, Japan) at 50 ng/mL and further incubated for 24 h. The collected culture media were used for TNF-α measurements.

### 4.10. ELISA for TNF-α

Mouse TNF-alpha DuoSet (R&D Systems, Minneapolis, MN, USA) and rat TNF-alpha DuoSet (R&D Systems, Minneapolis, MN, USA) were used to measure TNF-α levels in culture media and plasma according to the manufacturer’s instructions.

### 4.11. Measurements of AST and ALT

An aspartate aminotransferase colorimetric activity assay kit (Cayman Chemical Company, Ann Arbor, MI, USA) and alanine aminotransferase (ALT or SGPT) activity colorimetric/fluoro assay kit (Biovision Inc., Milpitas, CA, USA) were used to measure AST and ALT, respectively, according to manufactures’ instructions.

### 4.12. Measurement of Vascular Relaxation

Thoracic aortas were cut into rings of approximately 3 mm in length, and two tungsten wires were passed through the lumen of the rings and attached to the chamber filled with Krebs-Ringer solution of the 610M myograph system (Danish Myo Technology, Aarhus, Denmark). The Krebs-Ringer solution consisted of 111 mM NaCl, 5.9 mM KCl, 2.5 mM CaCl_2_·2H_2_O, 1.2 mM MgCl_2_·6H_2_O, 1.2 mM NaH_2_PO_4_·2H_2_O, 25 mM NaHCO_3_, and 11.5 mM D-glucose and was aerated with a 95% O_2_ and 5% CO_2_ atmosphere at 37 ℃. Isometric mechanical responses were recorded using a PowerLab 8/30 data acquisition system (AD Instruments, Bella Vista, NSW, Australia). The thoracic aortic rings were tensioned with 1.5 g resting tension and pre-contracted with phenylephrine (1 μM). Cumulative concentrations of acetylcholine (10^−9^ to 10^−5^ M) were added to the chamber, and tension was recorded. Finally, papaverine (100 µM) was added to induce maximum relaxation.

### 4.13. Statistical Analysis

All data are expressed as mean ± SD. Comparisons among the groups were analyzed by Student’s *t*-test, Williams test, or Tukey–Kramer test. A *p*-value of less than 0.05 was considered to indicate a statistically significant difference.

## 5. Conclusions

In conclusion, our findings demonstrate that ethanol extracts of *Saururus chinensis* (Lour.) Baill. (SE) could inhibit AGEs–RAGE interactions and subsequent NF-κB activation of RAGE downstream signaling, LPS-induced RAGE-dependent inflammatory reactions, and vascular endothelial dysfunction in diabetes. SE is a potent RAGE inhibitor for the prevention and treatment of RAGE-associated diseases such as inflammation and diabetes-associated endothelial dysfunction.

## 6. Patents

These results have been applied for a patent (patent application number: 2019-151641) in Japan.

## Data Availability

The data in this study are available from the corresponding author upon request.
